# Dynamic changes in *LINC00458*/*HBL1* lncRNA expression during hiPSC differentiation to cardiomyocytes

**DOI:** 10.1038/s41598-023-49753-3

**Published:** 2024-01-02

**Authors:** Patrycja Maciak, Agnieszka Suder, Jakub Wadas, Faith Aronimo, Paolo Maiuri, Michał Bochenek, Krzysztof Pyrc, Anna Kula-Pacurar, Marta Pabis

**Affiliations:** 1 Małopolska Centre of Biotechnology, Jagiellonian University, Gronostajowa 7A, 30-387 Cracow, Poland; 2 Virogenetics Laboratory of Virology, Małopolska Centre of Biotechnology, Jagiellonian University, Gronostajowa 7A, 30-387 Cracow, Poland; 3 Doctoral School of Exact and Natural Sciences, Jagiellonian University, Łojasiewicza 11, 30-348 Cracow, Poland; 4 Department of Molecular Medicine and Medical Biotechnology, University of Naples Federico II, Naples, Italy

**Keywords:** Cell biology, Stem cells

## Abstract

Long non-coding RNAs (lncRNAs) constitute the largest and most diverse class of non-coding RNAs. They localize to the nucleus, cytoplasm, or both compartments, and regulate gene expression through various mechanisms at multiple levels. LncRNAs tend to evolve faster and present higher tissue- and developmental stage-specific expression than protein-coding genes. Initially considered byproducts of erroneous transcription without biological function, lncRNAs are now recognized for their involvement in numerous biological processes, such as immune response, apoptosis, pluripotency, reprogramming, and differentiation. In this study, we focused on *Heart Brake lncRNA 1* (*HBL1*), a lncRNA recently reported to modulate the process of pluripotent stem cell differentiation toward cardiomyocytes. We employed RT-qPCR and high-resolution RNA FISH to monitor the expression and localization of *HBL1* during the differentiation progression. Our findings indicate a significant increase in *HBL1* expression during mesodermal and cardiac mesodermal stages, preceding an anticipated decrease in differentiated cells. We detected the RNA in discrete foci in both the nucleus and in the cytoplasm. In the latter compartment, we observed colocalization of *HBL1* with Y-box binding protein 1 (YB-1), which likely results from an interaction between the RNA and the protein, as the two were found to be coimmunoprecipitated in RNP-IP experiments. Finally, we provide evidence that *HBL1*, initially reported as an independent lncRNA gene, is part of the *LINC00458* (also known as *lncRNA-ES3* or *ES3*) gene, forming the last exon of some *LINC00458* splice isoforms.

## Introduction

Long non-coding RNAs are defined as transcripts that have no protein-coding function and are longer than 200 nucleotides, which distinguishes them from the other classes of non-coding RNAs, such as microRNAs (miRNAs), small nuclear RNAs (snRNAs), and small nucleolar RNAs (snoRNAs). Localized in intergenic regions (lincRNAs) or overlapping with protein-coding genes, they are predominantly transcribed by RNA polymerase II and undergo capping, splicing, and polyadenylation. When compared with protein-coding genes, lncRNAs are more numerous, less conserved evolutionarily, generally exhibit lower expression levels and are more developmental stage- and tissue-specific^1–4^. They are gaining increasing recognition as important regulators of various cellular processes, such as proliferation, apoptosis, stress response, pluripotency maintenance, and differentiation, through the control of gene expression at both the transcriptional and posttranscriptional levels^2,5^. LncRNAs are able to fold into complex three-dimensional structures, and to bind RNA, DNA and proteins, offering platforms for complex molecular assemblies. LncRNAs can localize within the nucleus, cytoplasm, or both, where they act by distinct mechanisms^6^.

In the nucleus, lncRNAs are involved in nuclear organization by contributing to nuclear body formation and chromatin organization. Nuclear lncRNAs act at the epigenetic level to modulate chromatin structure and function, at the transcriptional level to either suppress or activate the transcription of neighboring or distant genes, and at the posttranscriptional level to affect RNA splicing, stability or transport^7^. In the cytoplasm, lncRNAs can negatively regulate gene expression by inhibiting translation and mediating RNA degradation. They can also enhance gene expression by competing with miRNA binding sites on messenger RNAs or by sequestering and inactivating miRNAs acting as miRNA molecular sponges^1,6,8^. The latter ones, termed competitive endogenous RNAs (ceRNAs), illustrate an interesting interplay between lncRNAs and miRNAs. Apart from their physiological functions, lncRNAs are also involved in disease development, including cancer, cardiovascular disease and neurological disorders^9^.

Pluripotent stem cells (PSCs) are characterized by their exceptional capacity for self-renewal and the ability to differentiate into cells across all three germ layers. This makes them widely used in the stem cell field in the areas of disease modeling, drug discovery, and regenerative medicine^10^. Intricate mechanisms, which are in part contingent on long non-coding RNAs, underpin the pluripotency of these stem cells^6^. This is particularly relevant considering that many physiological pathways in stem cells are upregulated in cancer cells, thereby contributing to their tumorigenic properties. Hence, a comprehensive understanding of lncRNA functions within stem cell biology, covering both the preservation of pluripotency and the regulation of differentiation, is crucial not only for deciphering fundamental cellular mechanisms but also for exploring potential therapeutic applications and advancing our knowledge of cancer cell biology.

Genome-wide screens in pluripotent stem cells have identified many lncRNAs as essential components for maintaining pluripotency and driving differentiation. A recently reported lncRNA, *Heart Brake lncRNA 1* (*HBL1*), is involved in the regulation of the differentiation of human pluripotent stem cells toward cardiomyocytes (CMs) through counteracting microRNA-1 (*miR-1*) and guiding Polycomb Repressive Complex 2 (PRC2) chromatin occupancy^11,12^.

We set out our research to examine the progression of *HBL1* expression and its nuclear-cytoplasmic distribution pattern during the differentiation of human induced pluripotent stem cells (hiPSCs) into cardiomyocytes. First, our findings revealed that during CM differentiation, *HBL1* levels increased significantly during the mesoderm and cardiac mesoderm stages and subsequently decreased as the cells transitioned into cardiac progenitors. Second, utilizing RNA FISH, we showed that *HBL1* accumulated in discrete foci within the nucleus and cytoplasm, and that in the latter compartment it colocalized with the YB-1 protein. We observed that the number of *HBL1* foci initially increased and then decreased over the course of differentiation. Finally, we present evidence that *HBL1* is expressed as the 3′ end exon of *LINC00458* (also known as *lncRNA-ES3* or *ES3*), resulting from intricate alternative splicing occurring at this particular genomic location.

## Results

### *HBL1* expression peaks during the mesodermal stage of hiPSC differentiation to CMs

**Figure 1:**
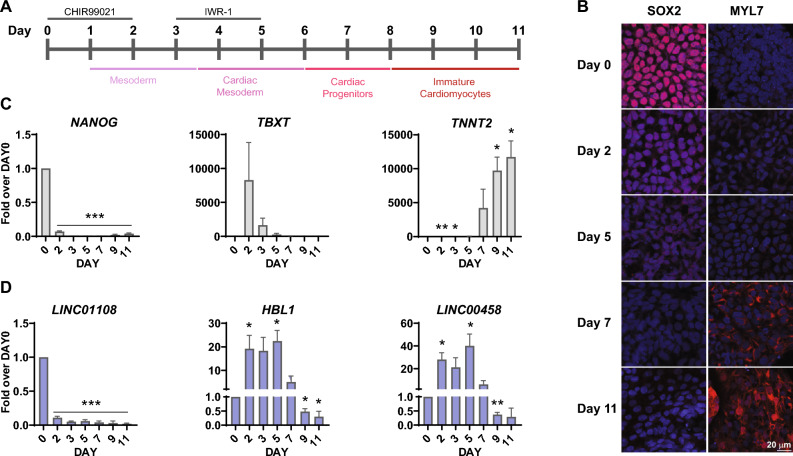
Expression profile of *HBL1* during hiPSC differentiation to cardiomyocytes. (**A**) Schematic representation of the small molecule-based differentiation protocol. Cells were incubated with the Wnt pathway activator CHIR99021 and, subsequently, the Wnt pathway inhibitor IWR-1. Beating cells started to appear around days 10–11 of differentiation. (**B**) Cells at selected points of differentiation were fixed and permeabilized, and immunofluorescence staining was performed to visualize selected markers of pluripotency (SOX2) and cardiomyocytes (MYL7). Scale bar = 20 µm. (**C**,**D**) RNA was purified from cells at the indicated timepoints and subjected to RT-qPCR analysis with primers amplifying (**C**) markers of pluripotency (*NANOG*), mesoderm (*TBXT*), and cardiomyocytes (*TNNT2*), as well as (**D**) selected lncRNAs. Data are shown as the mean ± standard deviation (n = 3). * p < 0.05, ** p < 0.01, *** p < 0.001, **** p < 0.0001, Student’s t-test, two-tailed, paired.

*HBL1*, a recently described lncRNA implicated in cardiogenesis, is present in human pluripotent stem cells but not in differentiated CMs (Day 20)^11^. We showed that its expression is indeed predominant in pluripotent stem cells compared with human umbilical cord-derived mesenchymal stem cells (UC-MSCs), human bone osteosarcoma epithelial cells (U2OS), and human embryonic kidney cells (HEK 293 T) (Supplementary Fig. S1) and wished to carefully monitor its exact temporal expression profile during hiPSC differentiation to CMs. We therefore applied a small molecule-based approach to differentiate cardiomyocytes from hiPSCs^13^ (Fig. 1A). The efficiency of differentiation was evaluated using immunofluorescence and flow cytometry. The pluripotency marker SOX2 decreased after differentiation initiation, while the expression of cardiomyocyte markers (cardiac troponin T (TNNT2) and atrial myosin light chain 2 (MYL7)) appeared on day 7, and their levels subsequently increased (Fig. 1B and Supplementary Fig. S2A). As assessed by flow cytometry, cells positive for both TNNT2 and MYL7 constituted 80% of the cell population on day 12 of differentiation (Supplementary Fig. S2B). Furthermore, RT-qPCR was applied to monitor the levels of selected differentiation markers and lncRNAs (Fig. 1C and D). *NANOG* was present in undifferentiated hiPSCs, and its levels decreased significantly at early differentiation timepoints (Day 2). The expression of *TBXT*, an early mesodermal marker, peaked on the second and third days of differentiation at the mesodermal stage. Finally, *TNNT2* appeared in cardiac progenitor cells, subsequently increasing its expression level (Fig. 1C). Having confirmed the efficiency of our differentiation protocol, we further applied RT-qPCR to monitor *HBL1* expression. We also included two other lncRNAs that are reported to be specifically expressed in pluripotent cells: *LINC01108* (*lncRNA-ES1*; *ES1*) and *LINC00458*^14^. We expected a steep decrease in their expression levels, similar to the pluripotency-specific expression of *NANOG* (Fig. 1C). The anticipated pattern was true only for *LINC01108*, while both *HBL1* and *LINC00458* significantly increased at the mesodermal stage of the differentiation process and decreased only when cells committed to the cardiac differentiation pathway (Fig. 1D). This decrease is clearly correlated with increased expression of cardiac-specific genes, e.g., *TNNT2* (Fig. 1C). To rule out the possibility that the observed expression is specific for the hiPSC line in use (established from endothelial progenitor cells, derived from peripheral blood), we subjected another hiPSC line (reprogrammed from peripheral blood mononuclear cells) to the same differentiation protocol. Despite the fact that differentiation efficiency appeared weaker in this cell line, as judged by *TBXT* and *TNNT2* expression levels, *HBL1* did exhibit a comparable expression profile in both cell lines (Supplementary Fig. S3). Thus, the peak of *HBL1* expression between days 2 and 5 of hiPSC differentiation to CMs is not an artifact of one specific hiPSC line. The exact biological significance and function, as well as mechanisms controlling this increased expression at the mesodermal stage prior to cardiomyocyte commitment, remain to be addressed in the future. It can be hypothesized that this expression pattern is related to the role of *HBL1* in guiding and maintaining PRC2 binding to cardiogenic genes, as the onset of cardiac gene expression (*e.g., TNNT2*) is concomitant with a decrease in *HBL1* levels (Fig. 1D), thus being in line with the proposed involvement of *HBL1* in cellular mechanisms controlling the maintenance (high *HBL1* expression) and subsequent derepression (low *HBL1* expression) of PRC2-mediated gene silencing of cardiogenesis-related factors^12^.

### Subcellular localization of *HBL1* in hiPSCs

**Figure 2:**
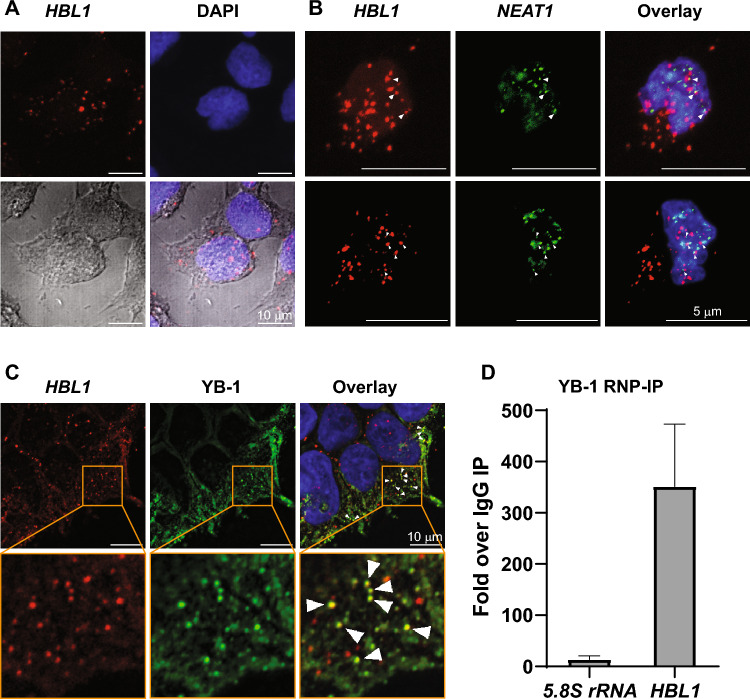
Subcellular localization of *HBL1*. (**A**) Human induced pluripotent stem cells were subjected to RNA FISH to detect *HBL1* lncRNA (shown in red). Nuclei were stained with DAPI (blue). Cells were visualized with transmitted light to appreciate the nuclear and cytoplasmic localization of *HBL1*. Scale bar = 10 µm. (**B**) On day 5 of differentiation toward cardiomyocytes, cells were subjected to double RNA FISH with probes targeting *HBL1* and *NEAT-1* lncRNAs. *HBL1* is shown in red, *NEAT-1* is shown in green, and DAPI-stained nuclei are shown in blue. Adjacent localization is shown by white triangles. Scale bar = 5 µm. (**C**) Human induced pluripotent stem cells were subjected to *HBL1* and YB-1 immuno-RNA FISH. *HBL1* is shown in red, YB-1 is shown in green, and DAPI-stained nuclei are shown in blue. Colocalization is shown by white triangles. Scale bar = 10 µm. (**D**) HiPSCs were collected and subjected to RNP-IP protocol using a Magna RIP^®^ RNA-Binding Protein Immunoprecipitation Kit according to the manufacturer’s instructions. A nonspecific IgG control was included in the experiment. Data are shown as the mean ± standard deviation (n = 3). P-values relative to IgG IP are: 0.1181 for *5.8S rRNA* and 0.0553 for *HBL1*, Student’s t-test, two-tailed, paired.

*HBL1* is present in both the nucleus and the cytoplasm of human pluripotent stem cells, as reported by Liu et al*.* using nuclear and cytoplasmic biochemical fractionation protocol^11^. We wished to assess its subcellular localization at the single-cell level in human induced pluripotent stem cells by taking advantage of RNA Fluorescence In Situ Hybridization assay (RNA FISH) and confocal microscopy. First, we optimized a protocol for *HBL1* lncRNA detection in human pluripotent stem cells using 20 sequence-specific probes recognizing *HBL1*. As shown in Fig. 2A, *HBL1* lncRNA exhibits punctate localization, forming foci that are scarcely distributed in the nucleus and the cytoplasm. The specificity of the FISH probes was validated in hiPSC-derived endothelial cells that do not express *HBL1* (Supplementary Fig. S4). The formation of *HBL1* foci may be driven by phase separation, as in the case of other membrane-free intracellular condensates composed of nucleic acids and proteins observed in both nuclei and the cytoplasm^15^. A similar localization pattern consisting of larger and smaller nuclear and cytoplasmic foci was also described for other lncRNAs, *e.g., ANCR* or *GAS5*^16^.

To further enhance our understanding of *HBL1*’s cellular functions and operational mechanisms, we aimed to explore whether there could be any overlap or interaction between *HBL1* and other cellular structures. As intracellular condensates are usually composed of nucleic acids and proteins, we focused on cellular bodies that contain proteins found among proteins interacting with *HBL1* in *HBL1* pulldown followed by mass spectrometry, *i.e.,* splicing factor, proline- and glutamine-rich (SFPQ) and Y box binding protein 1 (YB-1)^12^. We thus first focused our attention on paraspeckles, nuclear bodies that comprise, among others, the SFPQ protein and their structural component *NEAT1* lncRNA. Our interest in paraspeckles also stems from the fact that HBL1 function is related to chromosome modifications, and paraspeckles are reported to associate with genomic regions through *NEAT1* lncRNA^12,17^. To this end, we developed a double RNA FISH protocol to visualize both *HBL1* and *NEAT1* lncRNAs. We stained cells on day 5 of differentiation, as we expected to detect both *HBL1* foci, based on the high expression level of the transcript at this timepoint (Fig. 1D), and *NEAT1-*containing paraspeckles that form once cells exit the pluripotency state^18^. As shown in Fig. 2B, *HBL1* lncRNA appeared in both cellular compartments, while paraspeckles were exclusively nuclear, as expected. The majority of the nuclear *HBL1* lncRNA foci did not colocalize with *NEAT1* paraspeckles; however, we observed adjacent localizations between *HBL1* and *NEAT1* in a few cases. Further studies are needed to determine whether these RNAs bind overlapping chromatin sites within the nucleus. Moreover, it remains to be established whether any other nuclear bodies overlap with *HBL1* nuclear foci and to determine which other proteins or RNAs reside in *HBL1* nuclear foci. The first obvious candidates would be the PRC2 complex components.

Subsequently, we investigated the potential colocalization of *HBL1* with YB-1, a multifunctional RNA- and DNA-binding protein^19,20^. YB-1 is mainly localized in the cytoplasm and detected in small cytoplasmic mRNP granules, as well as in P-bodies (PBs) in unstressed cells and upon stress conditions in stress granules (SGs)^21–23^. YB-1 regulates apoptosis, cell proliferation, differentiation and stress responses and has been reported to be involved in several functional interactions with lncRNAs, such as *CAR10*, *GAS5*, *TMEM92-AS1*, *BASP-AS1*, *HOXC-AS3* and *HOTAIR*, some of which have been implicated in tumorigenesis^24–28^. Thus, given the presence of YB-1 in cytoplasmic bodies and reported functional interactions with lncRNAs, we wished to assess its potential interplay with *HBL1*. To this end, we used immuno-RNA FISH to visualize both YB-1 protein and *HBL1* lncRNA in pluripotent cells. As shown in Fig. 2C, YB-1 exhibited mainly cytoplasmic localization with some punctate staining in the cytoplasm. Interestingly, we observed colocalization between *HBL1* and YB-1 in the cytoplasm (Fig. 2C) in a subset of foci. We further wished to determine whether *HBL1* and YB-1 form ribonucleoprotein particles (RNPs), which we assessed by RNA immunoprecipitation (RIP) approach with anti-YB-1 antibodies. We detected an enrichment of *HBL1* in YB-1 IP compared with nonspecific IgG IP (Fig. 2D). The binding of YB-1 to highly abundant rRNAs was much lower, indicating a specific interaction between YB-1 and *HBL1*. While initially, the literature indicated a preference of YB-1 for A/C-rich regions^29,30^, the latest publications that applied CLIP and RNAcompete methods to study RNA–protein interactions indicate that YB-1 binds to a CU box (^C^/_G_CU^C^/_G_^C^/_U_^C^/_G_^A^/_U_) and a consensus motif CUGCG, respectively^31,32^. Nine CU box motifs can be found within the sequence of *HBL1* (Supplementary Fig. S5). To summarize, *HBL1* foci are distinct from paraspeckles and colocalize with YB-1-containing foci in the cytoplasm. It can be hypothesized that this colocalization is related to known *HBL1* interactions with miRNA-1 (miR-1) and Argonaute 2 (AGO2), the latter residing in P-bodies, sites of mRNA decay, where YB-1 is also detected in unstressed cells^11,22^. The functional significance of the *HBL1* interaction and colocalization with YB-1 will be pursued in the future.

### Visualization of *HBL1* lncRNA during hiPSC differentiation to cardiomyocytes

**Figure 3:**
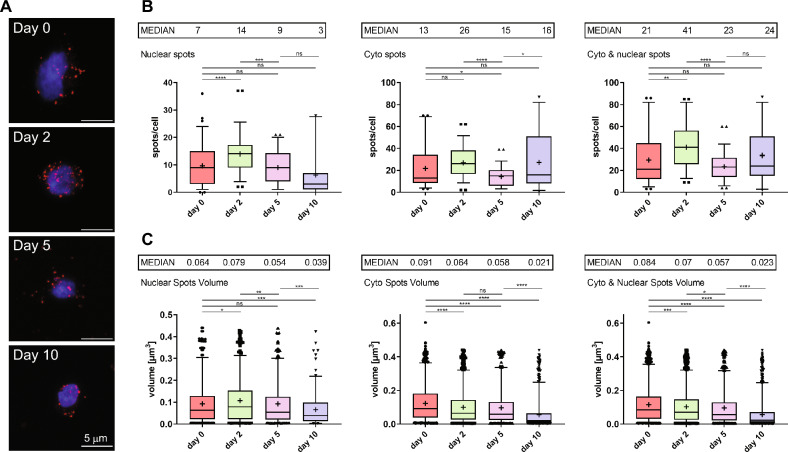
*HBL1* nuclear and cytoplasmic localization during hiPSC differentiation toward cardiomyocytes by RNA FISH. Human induced pluripotent stem cells were differentiated into cardiomyocytes over 10 days. On days 0, 2, 5, and 10, cells were collected and subjected to RNA FISH targeting *HBL1* lncRNA and analyzed using confocal microscopy. (**A**) Representative images of cells on days 0, 2, 5, and 10 as maximal projections reconstructed from confocal image stacks. *HBL1* RNA is shown in red, and DAPI-stained nuclei are shown in blue. Scale bar = 5 µm. The number (**B**) and volume [µm^3^] (**C**) of nuclear, cytoplasmic, and total (nuclear + cytoplasmic) *HBL1* lncRNA foci. Images of single cells were acquired with a confocal microscope, and *HBL1* RNA foci were quantified inside DAPI-stained nuclei and in the cytoplasm in z-stacks from 10 images/sample in three independent differentiations (n = 30). The results are presented as the mean values (depicted as “ + ”) ± s.d. Median values are shown as bars and indicated above each graph. Statistics were performed using the Welch two-sample t-test. Statistical comparisons are indicated if p < 0.05.

In parallel to cellular *HBL1* level measurement during differentiation using the RT-qPCR approach, the subcellular distribution of *HBL1* foci was addressed over time using our RNA FISH approach. Cells were collected at days 0, 2, 5 and 10, fixed, subjected to an RNA FISH protocol to detect *HBL1* lncRNA, and the number and volume of nuclear and cytoplasmic *HBL1* lncRNA foci were calculated (Fig. 3A). On day 0, we observed a median number of 7 condensates in nuclei and 13 in the cytoplasm (Fig. 3B). On day 2, we observed a twofold increase in the number of nuclear and cytoplasmic *HBL1* foci as compared to day 0 (median of 7 on day 0 and 14 on day 2 nuclear spots/cell and median of 13 on day 0 and 26 on day 2 cytoplasmic spots/cell). On day 5, the number of *HBL1* foci began to decrease when compared to day 2 (median of 9 nuclear spots/cell and of 15 cytoplasmic spots/cell). On day 10, we observed a further decrease in the number of nuclear spots (median of 3 nuclear spots/cell) and no changes in the number of cytoplasmic spots (median of 16 cytoplasmic spots/cell). Next, the volume of the nuclear and cytoplasmic *HBL1* foci was measured, which in general slightly decreased on day 2 and day 5 when compared to day 0, except for nuclear foci on day 2, where we observed an increase (Fig. 3C). On day 10, the volume of nuclear and cytoplasmic foci strongly decreased – we observed a 15- and 4.5-fold decrease compared to day 0, respectively. Altogether, we observed that the expression of *HBL1* peaked on day 2 of differentiation, as shown by the increase in the number of nuclear and cytoplasmic *HBL1* foci, and began to fall starting on day 5, with a strong decrease on day 10, as reflected by the drop in the number and volume of nuclear and cytoplasmic spots. Of note, cytoplasmic *HBL1* condensates did not change on day 10 compared to day 5, which suggests different stabilities of nuclear and cytoplasmic foci. In summary, the increase in *HBL1* lncRNA expression at the mesodermal stage (days 2 and 3) of differentiation correlates with increases in the number of nuclear and cytoplasmic foci (day 2), and the lower RNA abundance in cardiomyocytes (day 11) corresponds to a decrease in the number of nuclear foci and to a decrease in the volume of nuclear and cytoplasmic foci on day 10. Thus, the number and size of foci in general positively correlate with cellular levels of *HBL1*.

### Relationship of the *HBL1* gene with the* LINC00458* gene

**Figure 4:**
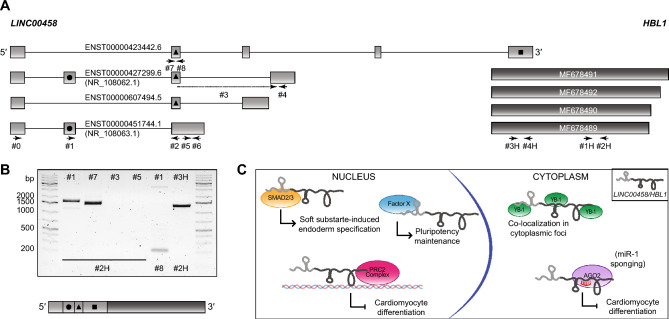
Relationship of the *HBL1* gene with the *LINC00458* gene. (**A**) Selected *LINC00458* isoforms according to GENCODE V39 12/2022 (light gray) and *HBL1* mRNAs from GenBank (dark gray). GENCODE transcript numbers and NCBI RefSeq numbers are indicated. The localization of primers is depicted by arrows and single digit numbers for *LINC00458* and H followed by single digit numbers for *HBL1*. Exons are represented as boxes, and introns are represented as lines. (**B**) RT-PCR products amplified with indicated primers selected such as to detect the presence of amplicons that encompass both *LINC00458* and *HBL1* sequences were separated by electrophoresis (upper panel). Forward primers are indicated at the top of the gel and reverse primers at the bottom. See Fig. 4A for the localization of the primers within the transcripts. PCR products amplified using primer #1 (as well as #0) combined with #2H were purified from the gel and subjected to Sanger sequencing, and the resulting sequence corresponded to the indicated *LINC00458* exons and *HBL1* (lower panel). (bp = base pairs). The original raw agarose gel image is presented in Supplementary Fig. S8A. (**C**) Model of *LINC00458/HBL1* cellular function proposed based on published data and our work. We propose that *LINC00458/HBL1* interacts with its binding partners and fulfils its diverse nuclear and cytoplasmic functions through separate functional domains encompassed within its sequence. The sequence corresponding to the 5′ exons of *LINC00458* is shown in light gray, and the 3′ end containing the *HBL1* sequence is shown in dark gray.

Finally, our attention was drawn by the similar expression profiles of *HBL1* and *LINC00458* observed during differentiation (Fig. 2D). *LINC00458* precedes *HBL1* on chromosome 13 and has multiple alternative splicing (AS) isoforms, some of which overlap with *HBL1* (Fig. 4A). With each new release of GENCODE annotations, the completeness and complexity of this locus increases (Supplementary Fig. S6A). This reflects a general trend for lncRNA annotation, resulting from a current drive in the GENCODE project to identify new transcripts and extend existing models based on the targeted incorporation of long-read datasets. *HBL1* was first described as an independent gene with 4 transcript variants differing in the length of their 3′ ends based on 5′ and 3′ RACE and PCR analysis with primers targeting selected sequences within *HBL1* and *LINC00458*^11^. However, through personal communication with Dr. Jonathan Mudge and Dr. Sílvia Carbonell Sala from the GENCODE Project, we established that long-read transcriptomics data clearly support *HBL1* being part of the same gene as *LINC00458*, constituting the last exon of some *LINC00458* AS isoforms. A subset of these longer isoforms is currently reported in the newest GENCODE release, V43 (Supplementary Fig. S6A). To further confirm these data, we amplified selected regions within *LINC00458* and *HBL1*, as well as amplicons starting within the *LINC00458* sequence and finishing at the *HBL1* 3′ end (Fig. 4B). We obtained PCR products for two amplicons encompassing exons located in the 5′ end of *LINC00458* and *HBL1* (primers #1 and #7 combined with primer #2H). No PCR products were detected for putative amplicons containing 3′ end sequences present in annotated AS isoforms of *LINC00458* (primers #3 and #5 combined with primer #2H). As positive controls, we amplified regions within *LINC00458* and *HBL1* (primers #1 combined with #8 and #3H combined with #2H, respectively). Additionally, we sequenced PCR products amplified by primers #0 and #1 combined with primer #2H (Fig. 4B and Supplementary Fig. S6B) and detected sequences of both *LINC00458* and *HBL1,* as depicted in Fig. 4B. The sequencing results were in agreement with the DNA molecule sizes estimated from the electrophoresis run. We thus conclude that the extremely complex *LINC00458* locus yields a variety of alternatively spliced isoforms, a subset of which encompasses the sequence of *HBL1*. These data do not completely rule out the possibility that *HBL1* exists as a separate gene. However, this possibility seems unlikely based on FANTOM5 CAGE data that do not support the presence of a transcription start site (TSS) for *HBL1* yet show a very clear TSS for *LINC00458* (Supplementary Fig. S7). In summary, based on the presented results and long-read transcriptomics data (from WTC-11 iPSC line), we postulate that *LINC00458* and *HBL1* constitute a single gene driven by the promoter region located immediately upstream of *LINC00458.*

## Discussion

In this study, we examined the expression and subcellular localization patterns of *Heart Brake lncRNA 1* during the differentiation of human-induced pluripotent stem cells into cardiomyocytes. Our findings can be summarized into the following key observations. First, the cellular levels of *HBL1* markedly increased during the mesoderm and cardiac mesoderm stages of differentiation, followed by a decrease at later stages of cardiac differentiation. Future research will investigate the functional implications of this expression pattern and the mechanisms driving its regulation. Second, we discovered that *HBL1* is localized in foci within both the nucleus and cytoplasm. The nuclear foci are distinct from paraspeckles, but adjacent localization is observed in some instances. This proximity may suggest an association with specific genomic regions necessary for gene expression control during development, a feature often associated with many lncRNAs^33^. We found that *HBL1* cytoplasmic condensates colocalize with YB-1, a multifunctional protein known to interact functionally with other lncRNAs, such as *HOTAIR*^25^. This colocalization might be related to *HBL1* interaction with *miR1* and AGO2^11^, all of which localize to cytoplasmic P bodies, or could be linked to others yet to be discovered cellular processes. Third, we uncovered that *HBL1*, initially characterized as a separate gene^11^, appears to be expressed as the last exon of *LINC00458*. We support this observation with several lines of evidence: (1) identical expression profiles of the two transcripts during differentiation, (2) detection of PCR amplicons spanning the sequences of both transcripts, (3) the presence of *LINC00458* isoforms that encompass the *HBL1* sequence in current GENCODE long-read transcriptomic data, and (4) FANTOM5 CAGE data indicating a transcription start site that clearly precedes the transcribed sequence of *LINC00458* but not *HBL1*. This said, we cannot fully exclude low levels of independent *HBL1* transcription from a minor transcription start site.

The observation that *LINC00458* and *HBL1* are part of the same gene raises the question how the distinct functions reported for the two transcripts, *i.e.,* pluripotency maintenance, as well as endodermal lineage specification for *LINC00458*, and control of cardiac differentiation for *HBL1*, can be reconciled. We propose that it could be achieved through the presence of distinct structural and functional domains within the same RNA, each serving a separate function. This concept aligns with the RNA modular code hypothesis, which suggests that lncRNAs possess multiple structural domains, each with its own unique function facilitated by specific interactions with RNA, DNA sequences, or protein complexes^34^.

In this context, the 5′ end of the transcript, which corresponds to the initially reported *LINC00458* sequence, might be involved in pluripotency maintenance interactions, although the mechanisms remain undefined^14^. Conversely, the 3′ terminal sequence of *HBL1* could regulate differentiation toward cardiomyocytes by counteracting *miR-1* and directing the chromatin occupancy of PRC^11,12^. Additionally, an expected but unidentified SMAD2/3 interaction site might relate to *LINC00458*’s role in substrate-specific endodermal lineage specification^35^. While the impact of *LINC00458* knockdown by siRNA or LNA GapmeRs on PSC differentiation into cardiomyocytes was not assessed^14,35^, the specific effect of *HBL1* on cardiomyocyte development, but not on stem cell pluripotency, was evaluated using CRISPR/Cas9 knockout of *HBL1*. This was achieved by targeting the sequence of *HBL1*, likely preserving the expression of shorter *LINC00458* isoforms^11^. Consequently, experimental data gathered in currently available literature do not rule out the possibility that *LINC00458/HBL1* might integrate the reported functions in pluripotency maintenance and modulation of differentiation by spatially separating interaction sites with specific binding factors within its sequence (Fig. 4C). This potential multifunctionality underscores the complexity and versatility of lncRNAs.

## Methods

### Cell culture and differentiation

A human iPSC line established from endothelial progenitor cells (derived from peripheral blood) was purchased from Reprocell and cultured on truncated recombinant human vitronectin (rhVTN-N) in Essential 8™ Flex medium. Cells were controlled for pluripotency level by flow cytometry analysis of Oct3/4 and Sox2 expression in cells and for mycoplasma by using the Venor GeM qEP kit. hiPSCs were differentiated toward cardiomyocytes based on the protocol published by Ref.^13^. Briefly, hiPSCs at 90% confluency were treated with 3 μM CHIR99021 in CDM3 medium (RPMI 1640 supplemented with 500 μg/mL recombinant human albumin and 213 μg/mL L-ascorbic acid 2-phosphate) for 48 h. After 24 h in CDM3 medium, the cells were treated for 48 h with 4 μM IWR-1 in CDM3 medium. Following 48 h in CDM3 medium, the cells were cultured in RPMI medium with B-27 supplement. Additionally, an alternative hiPSC line reprogrammed from peripheral blood mononuclear cells (PBMCs) was cultured and differentiated under the same conditions.

### RNA isolation, RT, qPCR, PCR

Total RNA was isolated using TRIzol™ Reagent, DNase-treated, and reverse-transcribed with a mixture of oligo(dT) reverse primer and random hexamers using an NG dART RT kit. qPCR was performed using SG qPCR Master Mix on a Quantstudio 6 Flex real-time PCR system (Thermo Fisher). The reactions were performed according to the manufacturer’s protocols. To calculate fold changes in mRNA levels, the following equation was used: 2^ΔCt(target)^/2^ΔCt(norm)^, where ΔCt = Ct (control sample) – Ct (experimental sample). Ct stands for threshold cycle value, “target” indicates the gene of interest, and “norm” indicates RNA for normalization. The cDNA amplified by conventional PCR with selected primers complementary to *HBL1* and *LINC00458* sequences was subjected to agarose gel analysis. Digital images of agarose gels were acquired using a ChemiDoc™ MP Imaging System (Bio-Rad) with Image Lab Touch Software (Bio-Rad), and bands selected for Sanger sequencing were cut out of the gel and purified using a GeneJET Gel Extraction Kit. See Supplementary Table S1 for primer sequences.

### Flow cytometry

Cells were harvested and dissociated using TrypLE™ Select Enzyme, fixed with 1% PFA for 20 min, permeabilized with 90% methanol for 10 min, and stained using 1:40 mouse monoclonal IgG1 TNNT2 and 1:200 mouse monoclonal IgG2b MLC2A (MYL7) for 1 h at RT. Mouse IgG1 and mouse IgG2b isotype controls were used. Secondary staining was performed with 1:1000 Alexa Fluor 488 goat anti-mouse IgG1 and 1:1000 Alexa Fluor 647 goat anti-mouse IgG2b for 30 min at RT. Cells were analyzed using a Navios (Beckman Coulter) flow cytometer. Data were analyzed using Kaluza 2.2 (Beckman Coulter) software.

### Immunofluorescence staining

Cells were fixed in 4% paraformaldehyde/PIPES for 10 min, permeabilized for 10 min with 0.5% Triton X-100, blocked in serum, and incubated with appropriate primary antibodies for 1 h at RT and fluorescent dye-conjugated secondary antibodies for 30 min at RT. Finally, coverslips were mounted in ProLong™ Gold Antifade Mountant with DAPI. Images were collected using an Axio Observer Z1 inverted microscope (Carl Zeiss, Jena, Germany) equipped with the LSM 880 AiryScan confocal module with ZEN 2012 SP1 black edition software and processed in ImageJ Fiji (National Institutes of Health, Bethesda, MD). A complete list of antibodies used is presented in Supplementary Table S2.

### RNA fluorescence in situ hybridization (RNA FISH) and confocal microscopy

hiPSCs and hiPSC-CMs on selected days of differentiation (days 0, 2, 5 and 10) were harvested, dissociated using TrypLE™ Select Enzyme, resuspended in an appropriate medium and plated on laminin-coated coverslips for 60 min at 37 °C. Next, the cells were fixed with 3.7% paraformaldehyde in 1 × PHEM (60 mM PIPES, 25 mM HEPES, 10 mM EGTA, 4 mM MgSO4·7H20) buffer for 10 min at RT. Next, cells were subjected to an RNA-FISH protocol using hybridization chain reaction (HCR) technology from Molecular Instruments, Inc. as previously described with modifications^36^. Briefly, cells were permeabilized with 0.1% Tween-20 in PBS for 10 min followed by incubation with a set of 20 DNA HCR v3.0 probes complementary to *HBL1* lncRNA (see Supplementary Table S3 for probe sequences) for 12 h at 37 °C. In the case of double RNA-FISH, cells were incubated simultaneously with two sets of probes complementary to *HBL1* ncRNA and *NEAT1* lncRNA. Next, cells were extensively washed and hybridized with HCR amplifiers labeled with Alexa 647 dye (for *HBL1*) and, in the case of double RNA-FISH, with additional amplifiers labeled with Alexa 488 dye (for *NEAT1*) for 12 h at RT in the dark and washed again. Finally, the nuclei were stained with 4′,6-diamidino-2-phenylindole (DAPI) (D1306; Thermo Fisher Scientific), and the cells were mounted on slides with Prolong Diamond Antifade Mounting Medium (P36970; Invitrogen). Fluorescence images were acquired using an Axio Observer Z1 inverted microscope (Carl Zeiss, Jena, Germany) equipped with the LSM 880 AiryScan confocal module with ZEN 2012 SP1 black edition software and processed in ImageJ Fiji (National Institutes of Health, Bethesda, MD). Extrapolated data were analyzed and plotted with R [R Core Team (2022). R: A language and environment for statistical computing. R Foundation for Statistical Computing, Vienna, Austria. URL https://www.R-project.org/ and Wickham H (2016). ggplot2: Elegant Graphics for Data Analysis. Springer-Verlag New York. ISBN 978-3-319-24277-4, https://ggplot2.tidyverse.org].

### Immuno-RNA FISH

HiPSCs were fixed using 3.7% PFA, washed thoroughly with PBS and permeabilized for 10 min using 0.1% Tween-20 in PBS (PBST) solution. The cells were then incubated for 30 min at 37 °C in probe hybridization buffer. Next, coverslips were put into a hand-made humidity chamber to incubate with *HBL1* lncRNA-specific probes overnight at 37 °C. Cells were washed 4 times with probe wash buffer preheated to 37 °C and 2 times with 5 × SSCT buffer. Next, the cells were incubated with snap-cooled Alexa 647-labeled hairpin amplificators overnight at room temperature in the dark. Cells were then washed 5 times with 5 × SSCT buffer, blocked in 5% BSA in PBST solution for 2 h at room temperature and subjected to rabbit α-YB-1 (1:200) antibody incubation in 1% BSA and 0.02% Tween-20 in PBS at 4 °C overnight. The next day, the cells were washed using PBST buffer 3 times, incubated with goat α-rabbit Alexa 594 antibodies (1:400) in PBS for 2 h at RT and then washed 3 times using PBST buffer. Finally, the nuclei were stained with DAPI in PBS for 10 min at room temperature and subjected to confocal microscopy analysis. See Supplementary Table S2 for antibody information.

### Immunoprecipitation of ribonucleoproteins (RNP-IP)

The assay was performed using a Magna RIP^®^ RNA-Binding Protein Immunoprecipitation Kit according to the manufacturer’s instructions. The isolated RNA was DNase-treated and subjected to reverse transcription. Fold enrichment of a specific antibody-precipitated RNA over nonimmune IgG IP was determined by quantitative real-time PCR (qPCR). The following equation was used: 2^(Ct(IgG)—Ct(Spec))^, where Ct stands for threshold cycle value, IgG for nonimmune IgG IP and Spec for specific antibody IP.

### Supplementary Information

Supplementary Information.

## Data Availability

The authors confirm that the data supporting the findings of this study are available within the article and its supplementary materials.
