# Supplementary material for: Dynamic changes in LINC00458/HBL1 lncRNA expression during hiPSC differentiation to cardiomyocytes
Source: Sci Rep. 2024 Jan 2;14:109. doi: 10.1038/s41598-023-49753-3 (PMC10761834; doi:10.1038/s41598-023-49753-3)
Supplement: Supplementary file 1 — Supplementary Information. [file 41598_2023_49753_MOESM1_ESM.pdf]

SUPPLEMENTARY INFORMATION

Dynamic changes in *LINC00458*/*HBL1* lncRNA expression during hiPSC differentiation to cardiomyocytes

Patrycja Maciak[#,1], Agnieszka Suder[#,2,3], Jakub Wadas[2,3], Faith Aronimo[1], Paolo Maiuri[4], Michał Bochenek[1], Krzysztof Pyrc[2], Anna Kula-Pacurar[*,2], Marta Pabis[*,1]

# Equal contribution

Affiliations:

[1] Malopolska Centre of Biotechnology, Jagiellonian University, Gronostajowa 7A, 30-387 Cracow, Poland.
[2] Virogenetics Laboratory of Virology, Malopolska Centre of Biotechnology, Jagiellonian University, Gronostajowa 7A, 30-387 Cracow, Poland.
[3] Doctoral School of Exact and Natural Sciences, Jagiellonian University, Lojasiewicza 11, 30-348, Cracow, Poland
[4] Department of Molecular Medicine and Medical Biotechnology, University of Naples Federico II, Naples, Italy.

* Correspondence to: Marta Pabis; email: marta.pabis@uj.edu.pl; Anna Kula-Pacurar; e-mail: anna.kula-pacurar@uj.edu.pl

Supplementary Table S1. Primers used in this study

**Target**	**Forward**	**Reverse**
GAPDH 1+2	GTGGACCTGACCTGCCGTCT	GGAGGAGTGGGTGTCGCTGT
RPS29 1+2	AATATGTGCCGCCAGTGTTT	CCCGGATAATCCTCTGAAGG
18S 1+2	TTGTTGGTTTTCGGAACTGAG	GCAAATGCTTTCGCTCTGGTC
5.8 S 1+2	CGACTCTTAGCGGTGGATCA	GGGCCGCAAGTGCGTTCGAA
5S 1+2	GTCTACGGCCATACCACCCT	AAAGCCTACAGCACCCGGTA
NANOG	TGAACCTCAGCTACAAACAGGTG	AACTGCATGCAGGACTGCAGAG
TBXT 3+4	CAAATCCTCATCCTCAGTTTG	GTCAGAATAGGTTGGAGAATTG
TNNT2 1+2	GGCAGCTCCTGTTTGGAAATG	TTATTACTGGTGTGGAGTGGGTGTG
HBL1 1 1+2	GGTTGTGGTAATGAGCTGGGA	TATGCTCTGCAGCCACTAGAC
HBL1 1 3+4	GCAGTCCTACACCTTGCCTT	TGGGAGTTGCAGGTGATGTC
HBL1 1 7+8	TGCACATTTTGAGGGGATAATTGG	GAAGGAACAAAGACACACTGCT
ES1 1+2	AGCAGAGACAGAATGAGCAATG	GCCGCAGATAAGCAAAATGAC
ES1 3+4	TGTTGGGAGCATTCTTTTTCTT	GCAGCATCTTTGTTGAGGTGTG
ES3 1+2	AAGACTGACACTGCCCAATCG	GCTGTAGGAAGGTTGTGAGATG
ES3 3+4	ACTGTGAGAACTCAAAGGGGG	ACGCCAACTAAGCAGACGTA
ES3 5+6	CAACATGAGTCATTGGGGCA	AATCTGTCTTTGTGGCAGGC
ES3 7+8	GGACTTTTTCTTCTGGACTGAAC	ACTTGCTGTAGGAAGGTTGTGA
*ES3*_0	GCCCAAGGAACATCTCACCA	

Supplementary Table S2. Reagents used in this study

Reagent	Source	Identifier
hiPSC	Reprocell	Cat # RCRP005N
rhVTN-N	Thermo Fischer Scientific	Cat # A31804
Essential 8™ Flex medium	Thermo Fischer Scientific	Cat # A2858501
RPMI 1640	Thermo Fischer Scientific	Cat # 11875
L-ascorbic acid 2-phosphate	Sigma-Aldrich	Cat # A8960
Albumin Human, Recombinant	Sigma-Aldrich	Cat # A9731
CHIR99021	Selleckchem	Cat # S1263
IWR1	Sigma-Aldrich	Cat # I0161
B-27 supplement	Thermo Fischer Scientific	Cat # 17504-044
TRIzol	Thermo Fischer Scientific	Cat # 15596026
TURBO™ DNase	Thermo Fischer Scientific	Cat # AM2238
NG dART RT kit	EURx	Cat # E0801-02
SG qPCR Master Mix	EURx	Cat # E0402-03
PMSF Solution (0,1 M in Ethanol)	Sigma-Aldrich	Cat # 93482
Pierce™ BCA Protein Assay Kit	Thermo Fischer Scientific	Cat # 23225
Formaldehyde solution	Sigma-Aldrich	Cat # F8775
Triton X-100	Sigma-Aldrich	Cat # T8787
ProLong™ Gold Antifade Mountant with DAPI	Thermo Fischer Scientific	Cat # P36941
GeneJET Gel Extraction Kit	Thermo Fischer Scientific	Cat # K0691
TrypLE™ Select Enzyme	Thermo Fischer Scientific	Cat # 12563029
ECMatrix™-511 Silk E8 Laminin	Sigma-Aldrich	Cat # CC161
Magna RIP® RNA-Binding Protein IP Kit	Sigma-Aldrich	Cat # 17-700
Venor®GeM qEP	Minerva Biolabs	Cat # 11-9025
GeneRuler 1 kb Plus DNA Ladder	Thermo Fischer Scientific	Cat # SM1331
HCR Probe Hybridization Buffer	Molecular Instruments, Inc.	Cat # BPH03821
HCR Probe Wash Buffer	Molecular Instruments, Inc.	Cat # BPW01522
HCR Amplification Buffer	Molecular Instruments, Inc.	Cat # BAM01522
HCR amplifier B1-h1 AF 647	Molecular Instruments, Inc.	Cat # S013922
HCR amplifier B1-h2 AF 647	Molecular Instruments, Inc.	Cat # S012522
**Antibodies**	**Source**	**Identifier**
anti-SOX2 (rabbit)	Abcam	Cat # ab97959
anti-TNNT2 (mouse IgG1)	Thermo Fischer Scientific	Cat # MA5-12960
anti-MYL7 (mouse IgG2b)	Synaptic Systems	Cat # 311011
anti-rabbit IgG, Alexa 647 (donkey)	Jackson Laboratories	Cat # 711-606-152
anti-mouse IgG2b, Alexa 647 (goat)	Thermo Fischer Scientific	Cat # A-21242
anti-mouse IgG1, Alexa 488 (goat)	Thermo Fischer Scientific	Cat # A-21121
Mouse IgG1 Isotype Control	Thermo Fischer Scientific	Cat # MA1-10406
Mouse IgG2b Isotype Control	Thermo Fischer Scientific	Cat # MA1-10427
anti-YB-1 (rabbit)	Sigma-Aldrich	Cat # Y0396
anti-rabbit IgG, Alexa 594 (goat)	Thermo Fischer Scientific	Cat # A-11012

Supplementary Table S3. *HBL1* FISH probes used in this study

**1**	ACACTGCCAAGTCTCCCTAATGTCAACAAGGCTAGCAGTCATTCCTGGGCCA
**2**	TTGTTTGCTTGAATTACCTCTTTGGGACGTGAGGAATTTACACTAAAACACC
**3**	TTTCTTTACAGAAAAGATACTAGCGCCAGTCTGGCAGGGGCCTTTTCTAGTC
**4**	TGCCCTAACATGGAGTCCAAATTTCGTTAGCATGGTTTAAGAGTCTCTTCAT
**5**	GACCACAAAACCACAGTCCAGATTGTAGGAGGCTTGGGTTGTGGTAATGAGC
**6**	CTGCATCAATCAGGACATCAGTGTTACTGATTTGCGAGACTAGGGAATTAGG
**7**	TCATCATTATTACTAAGCAGTCCTATGTTAATAGCCCTTTGGGTCTAGAGCT
**8**	AATTATGCGCTATACCTTGTACTTGAAAGAAACTATATCCTGACTAGTCCAG
**9**	AAGCTACCTGGACTTCAATATTACTCTTGTGACCCGAATTATCTTATTTAGT
**10**	TTTTCTTGTTTGGTGCAAGTCAGCTCCTGGAAGGTGGAAGTGTTTTGTTCTC
**11**	TAATACCTGCCTTCAAGAAACTAACTCTCAAGGCTGCTGTATGGAAATCCTG
**12**	GGACTTCTCTGCTGGCTATCAGGAGTCCTCATCTATGATCTGCTAGAAGAAT
**13**	GTTCTTGATGAAAGTGAGCAAAGGAATTTTGCAGAGATCAGAGTAGAACTTT
**14**	CAGCCTCCTGAAGCCTGGCAAGGGTGCCTTAGTGAGGACATCACCTGCAACT
**15**	TCCTAACTAGATTGCACTCTTCTAAATGCTGTACTACTGCTATACACATTCT
**16**	CATGTGTACGTCTTTCCATTCCTGCATCCCAGGGGATTGCAGCCATCAGCTC
**17**	CCAAGAAAGGTCCCCTTCCTAGCAACTGTGCATCCTTCCAGCTGCCTTATCA
**18**	TTGCATCTCCTGGGATTTTTGCCATAGGCTTTTCTGTACTGTTTGCATTTCT
**19**	AAGCACGCTGAGCAGTCCTACACCTTGCCTTCTGAGAGAGAAGAGGACAGTC
**20**	GCAAATACTGTGAACGCTGTCACACTAGCACAAAGCCTTTGCAAAAATTTCC

Supplementary figures and figure legends

SUPPLEMENTARY FIGURE S1

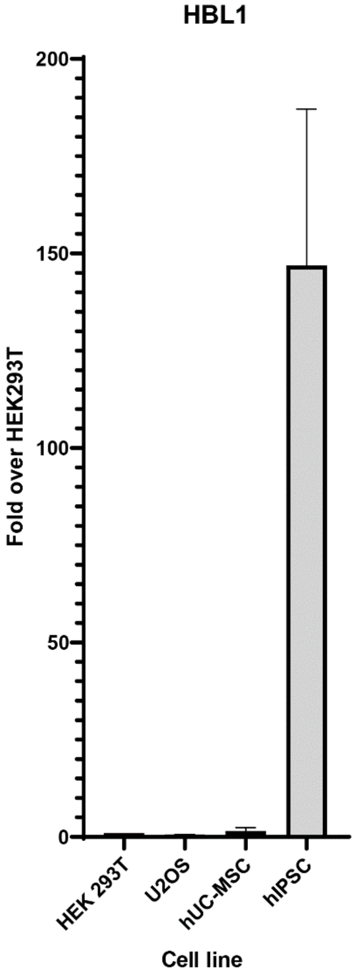

SUPPLEMENTARY FIGURE S1. Relative expression levels of *HBL1* in selected human cell lines. RNA from HEK 293T, U2OS, hUC-MSC and hiPSC was isolated, DNase-treated and subjected to RT-qPCR analysis with primers amplifying an amplicon within *HBL1* (primers 1+2). Data are shown as mean ± standard deviation (n = 2).

SUPPLEMENTARY FIGURE S2

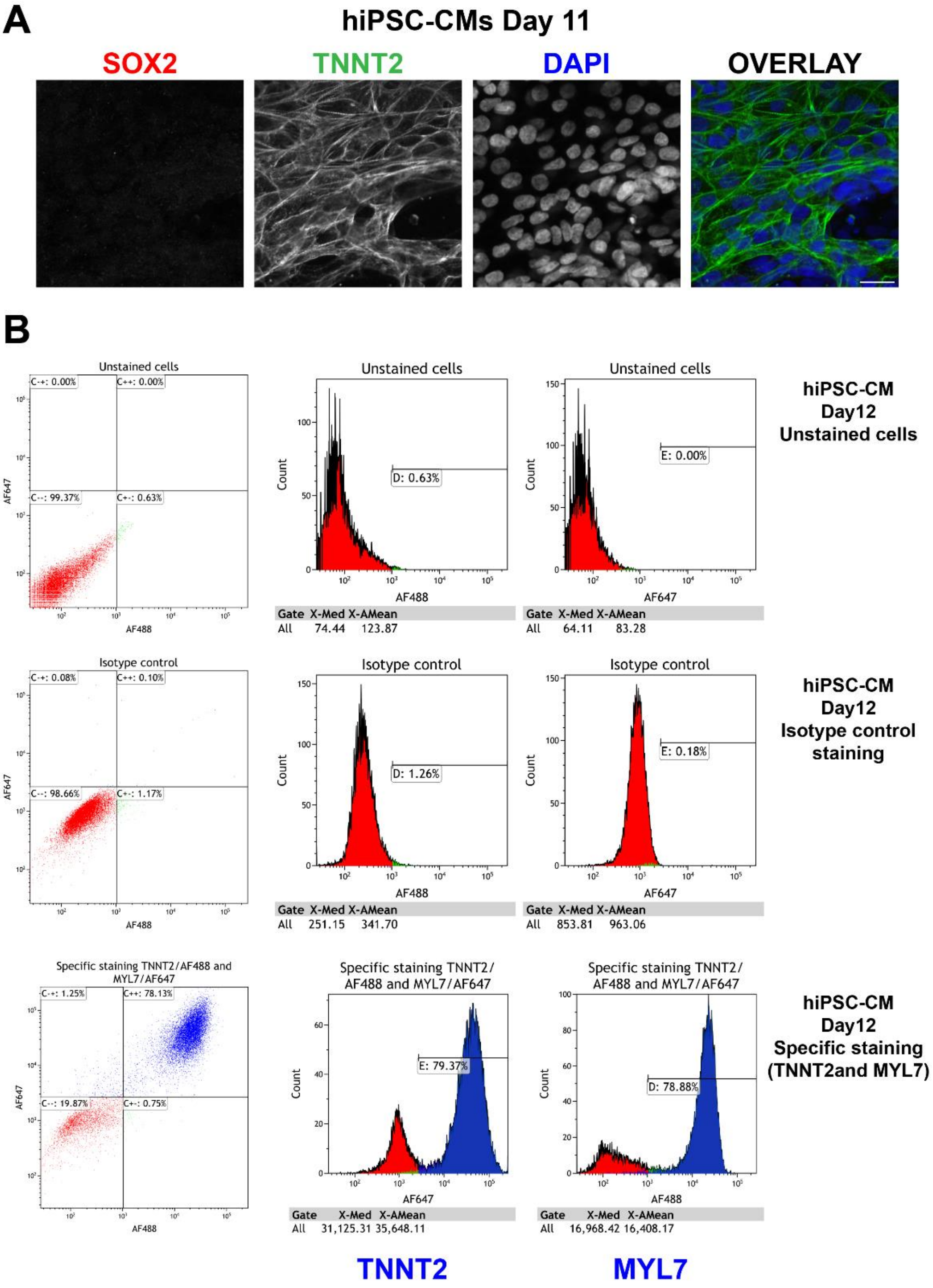

SUPPLEMENTARY FIGURE S2. Validation of hiPSC differentiation towards cardiomyocytes. Cells at selected points of differentiation were analysed regarding the presence of expected cardiomyocyte markers using confocal microscopy (**A**) and flow cytometry **(B)**. (**A**) Cells were fixed, permeabilized and immunofluorescence staining was performed to visualize both a markers of pluripotency (SOX2) and of cardiomyocytes (TNNT2). Scale bar, 20 µm. (**B**) Cells were dissociated, fixed, permeabilized and stained for flow cytometry using anti-TNNT2/AF488 and anti-MYL7/AF647 antibodies. Unstained cells and adequate isotype controls were included in the analysis.

SUPPLEMENTARY FIGURE S3

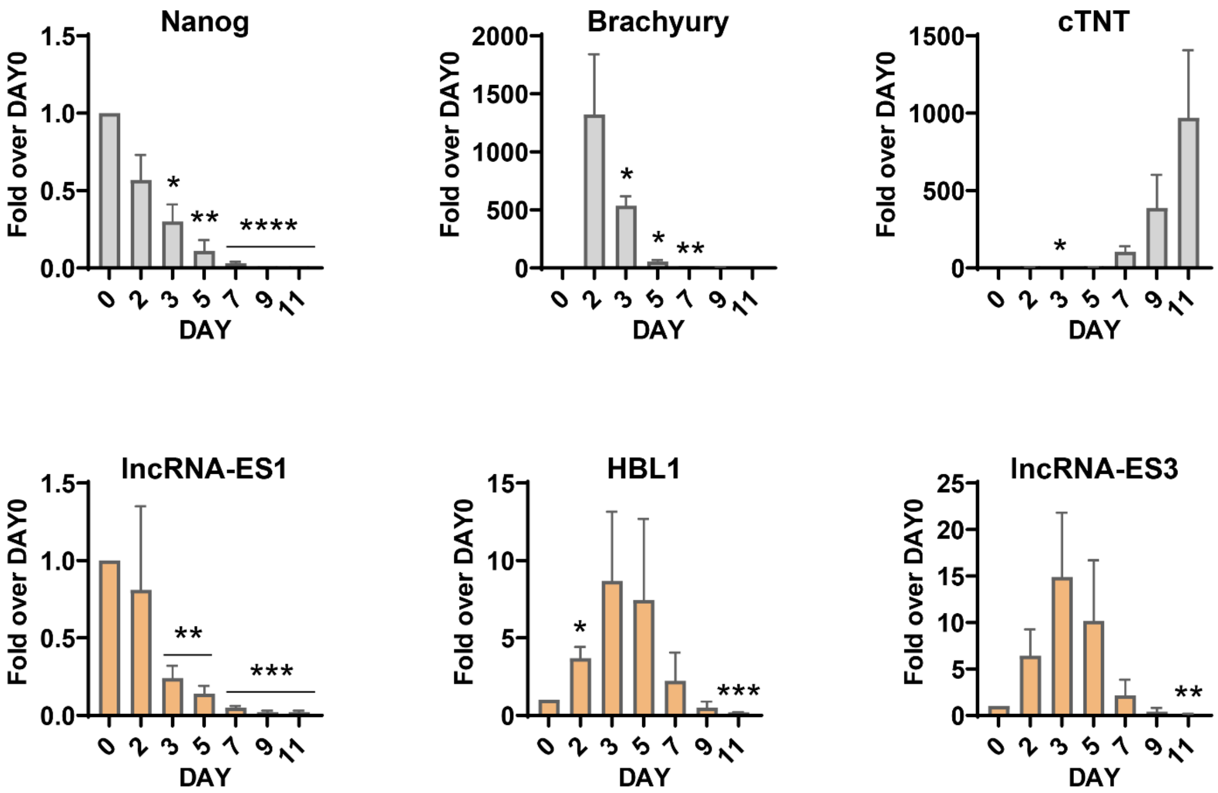

SUPPLEMENTARY FIGURE S3. Expression pattern of selected transcripts during differentiation of hiPSC (reprogrammed from PBMC) towards cardiomyocytes. RNA was purified from cells at indicated timepoints and subjected to RT-qPCR analysis with primers amplifying markers of pluripotency (*NANOG*), mesoderm (*TBXT*) and cardiomyocytes (*TNNT2*), as well as selected lncRNAs. Data are shown as the mean ± standard deviation (n = 3). * $p < 0.05$, ** $p < 0.01$, *** $p < 0.001$, **** $p < 0.0001$, Student's t-test, two-tailed, paired.

SUPPLEMENTARY FIGURE S4

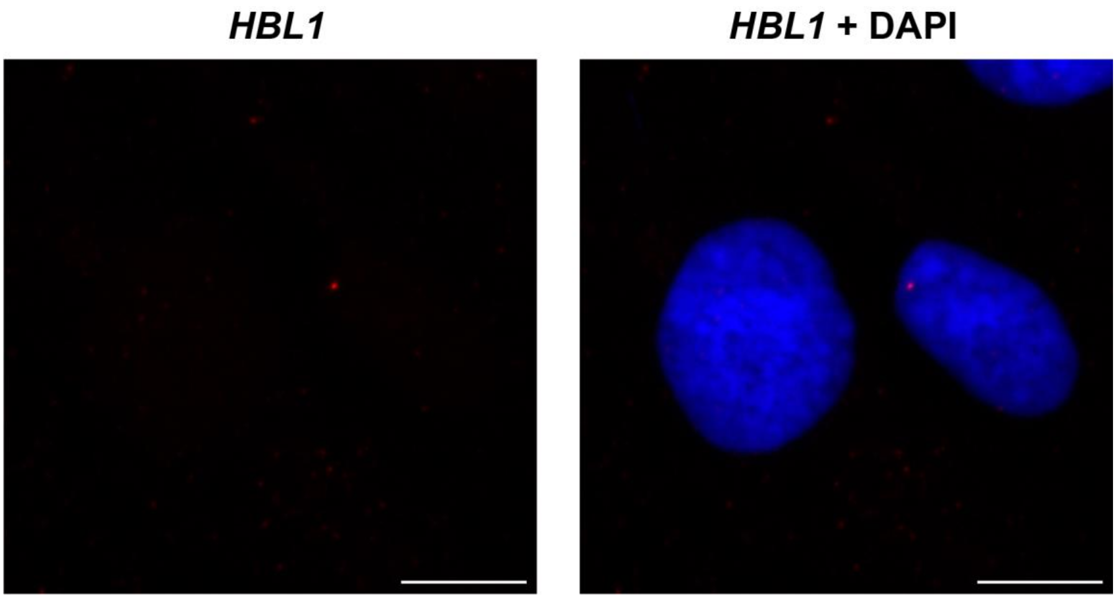

SUPPLEMENTARY FIGURE S4. hiPSC-derived endothelial cells were subjected to RNA FISH against *HBL1* lncRNA (left panel). Nuclei were stained with DAPI (in blue, right panel). Scale bar = 10 µm

SUPPLEMENTARY FIGURE S5

ACACTGCCAAGTCTCCCTAATGTCAACAAGGCTAGCAGTCATTCCTGGGCCAAATTTGTCCCCTTC
CTGAAGCAATGTCTGAAGCTGAACAGGAGTCACTCCCCAGCATCTCCCCAGTCCTTGTGTGCCAC
GTGGATAAAGAAGATACAGTCCCCATGACTGGCTAGCTTGAAGATGCCAACAGGTCTCAATTGTCT
TGACTTGGTTTATGACAAACCCAGCCTAAGGTTGCCATCTAAAAATGGAGACATGGCCGGACTCAT
CTTTCTCAAGCTACCTGGACTTCAATATTACTCTTGTGACCCGAATTATCTTATTTAGTTCTTTTCTTG
TTTGGTGCAAGTCAGCTCCTGGAAGGTGGAAGTGTTTTGTTCTCCATTGCATCTCCTGGGATTTTT
GCCATAGGCTTTTCTGTACTGTTTGCATTTCTGGTTGTTTGCTTGAATTACCTCTTTGGGACGTGAG
GAATTTACACTAAAACACCTGTTTCTTTACAGAAAAGATACTAGCGCCAGTCTGGCAGGGGCCTTT
TCTAGTCTCAAGCACGCTGAGCAGTCCTACACCTTGCCTTCTGAGAGAGAAGAGGACAGTCCTCA
GCCTCCTGAAGCCTGGCAAGGGTGCCTTAGTGAGGACATCACCTGCAACTCCCAGATGGATAGAC
GAGGCCATGGAGAGAGGAGAACACCAACCCATGAGTGACAAGTG**CCTGTCA**TTGTCAATGATGCT
GCAAGCACCACGATGCCCCTTCTTAGCAGGGACCCAGTGGGCCTTACCAGCTTCATTATTTTCTAG
GCATGATCGCAT**CCTGTCT**CCCATTTTTGCTAAAATTCTATCTAGAGAAAGAGCTCATTTTTAGCTAA
AAAAAAAAAAATCCTGTTTTATCTGCTAAAAAATCACATAAAATAACTATTGGACTGTTTGAAATGGC
GTGGCCTCT**GCTCTGA**CTTATTAACTGTGATCTTGACTAAGTTAAGCAGTCACTCTGACCTTACGTT
ACTCCCTCAAATTTGAATAAAAATAGCAACTTCCTCTACTTATAAATTACTTCTAAAAATTAGATTCTT
GAGATTCAAAGATAGGTAATA**CCTGCCT**TCAAGAAACTAACTCTCAAGGCTGCTGTATGGAAATCCT
GCCTCCTAACTAGATTGCACTCTTCTAAATGCTGTACTACTGCTATACACATTCTCCAAATCAAGTTA
ATTTCAAAGTCTTGCTGAATCTACTTTTTAGAAATATCTAAGTCATGTGTACGTCTTTCCATTCCTGC
ATCCCAGGGGATTGCAGCCATCAGCTCACCTCCTCTCCTAATCCAAAATGTTCCTAACTGGTCT**CC**
TGCCTTGCCTTTCTCCTGATCACTTCCTACAACAGAGTGATCGTGACTTGAAATGCAAATTATATTG
TATGAGTTATCTCCCAAAAACATTCAGCTGCCTA**GCTCCCT**TAACATGAAGTCCAAATTGCCCTAAC
ATGGAGTCCAAATTTCGTTAGCATGGTTTAAGAGTCTCTTCATGAACAAAAGGCTTCTGTCTTCTCC
AGTCATACCCAAATCTTTCCTAATCAAAAGCCATTTTTATCAGTTTAAGAACAGAGACAGTTGTTCCA
GATATAAGGAAGAGCATTGGCAAAGTTGCAGATAATAATTTAGGTGAGTGTGATTCAGAGATCTCAT
TAGTGATGCCTCAGAATGACTAGGAGACATGGATAAAAGGATAACAAGAAAGAAAAGGAGACCACA
AAACCACAGTCCAGATTGTAGGAGGCTTGGGTTGTGGTAATGAGCTGGGACTTCTCTGCTGGCTA
TCAGGAGTCCTCATCTATGATCTGCTAGAAGAATATCATGATTAGAACTGCATTTTAGAAAGTTAAGT
CTAGTGGCTGCAGAGCATACATATAGAAGGTAGAGCTTTGAGTCCACAAGAACATTATGGTTTAATA
AAACTCTGTGAGAGAGATTAGAGCACAAACAAAGGCAGTCATAATGGAAATGGAAAGAAAGTTAAA
GGCTAAAGACTCCAGAGACATTTTGAAAAATGTGGGAAATTAGGAGAGGAGCTGCATCAATCAGG
ACATCAGTGTTACTGATTTGCGAGACTAGGGAATTAGGATGTTCTTGATGAAAGTGAGCAAAGGAA
TTTTGCAGAGATCAGAGTAGAACTTTTTTTTTTCCCTGAAAGCATTCTGAAAACTGTAAACACCCTA
CTGCATAAGATATCATCATCATTATTACTAAGCAGTCCTATGTTAATAGCCCTTTGGGTCTAGAGCTT
GAATTATGCGCTATACCTTGTACTTGAAAGAAACTATATCCTGACTAGTCCAGATGGATATTACTTAA
CCTGCTTTAAGATCCTCTAGGGAGGAACAACCACAACAAATCCAAGAAAGGTCCCCTTCCTAGCAA
CTGTGCATCCTTCCA**GCTGCCT**TATCAATGATATCATCCCTTCGATATGTTACAGAGTCCATTTGGA
CAATTTTTAAAGGGACTCATGACATTCTGGTAAAAGAGAATTATGATTTCTGGACTCTTTAAAATTCA
AATGTAGGGGGAAAAAAAGCAAGTCTTCTGTTTGATCATAAAAGCTATAAAGATGCAAAGAGAGAAA
GGTCCATTCTAGCATTTCAATAATATCTGTTTTCACTGAAAACCTGAGCTGACTTTAATCATCTACAC
TTCAGACTGGGCTAATACCTCAGAAAGCTGCAAATACTGTGAAC**GCTGTCA**CACTAGCACAAAGCC
TTTGCAAAAATTTCCCCAAAATACACAGATTAAAAAGCACATTTTCTCTCTCTCTGTTTT**CCTCTCT**C
TCTCTCTCTCTCTCACACGCACACACACATACACATGCACAAAAATTTGTCATTAATTGTTAAATTAT
TTTCATTGTAGGAAAGATAAATCTTTCAATTAATATAGTATTGTTCATGCTAAAAAATGTCTTATCCATT
AAAAAGATACTCAGTTGTAATAGAGTAGTTCAATCAATACTGGTAAATTGCACATTTTGAGGGGATAA
TTGGTAAATGCAAACTCAACTAGAAAAGAAGAGAAAGCAGTGTGTCTTTGTTCCTTCTGT

SUPPLEMENTARY FIGURE S5. Sequences corresponding to CU box ($^{C}/_{G}CU^{C}/_{G}{}^{C}/_{U}{}^{C}/_{G}{}^{A}/_{U}$) are marked in orange on *HBL1* T3 sequence (MF678491).

SUPPLEMENTARY FIGURE S6

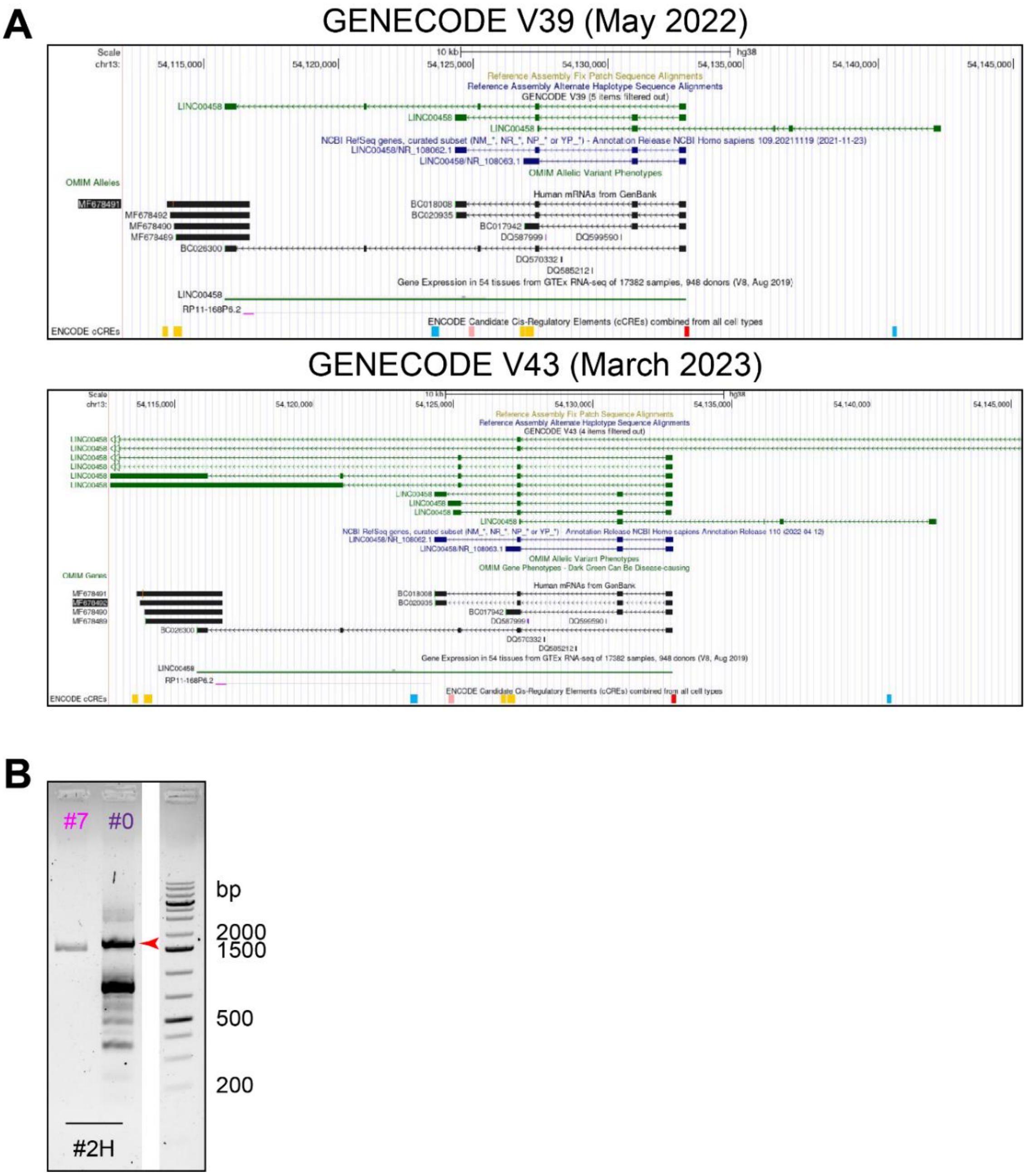

SUPPLEMENTARY FIGURE S6. (**A**) Genomic localization of *LINC00458/HBL1* from public databases. Screen shots from UCSC Genome browser (https://genome.ucsc.edu) presenting the information about the genomic locus encompassing *LINC00458* and *HBL1*, as well as the annotated splice isoforms of *LINC00458*. Two GENCODE releases are selected to present the increasing complexity of the locus identified based on annotations relying on long-read data (**B**) RT-PCR products amplified with indicated primers selected such as to detect the presence of amplicons that encompass both *LINC00458* and *HBL1* sequences were separated by electrophoresis. Forward primers are indicated at the top of the gel and reverse primers at the bottom. See Figure 4A for the localization of the primers within the transcripts. The PCR product indicated with the red arrowhead amplified using primer #0 combined with #2H was purified from the gel and subjected to Sanger sequencing. The original raw gel image is presented in Supplementary Fig. S8B.

SUPPLEMENTARY FIGURE S7

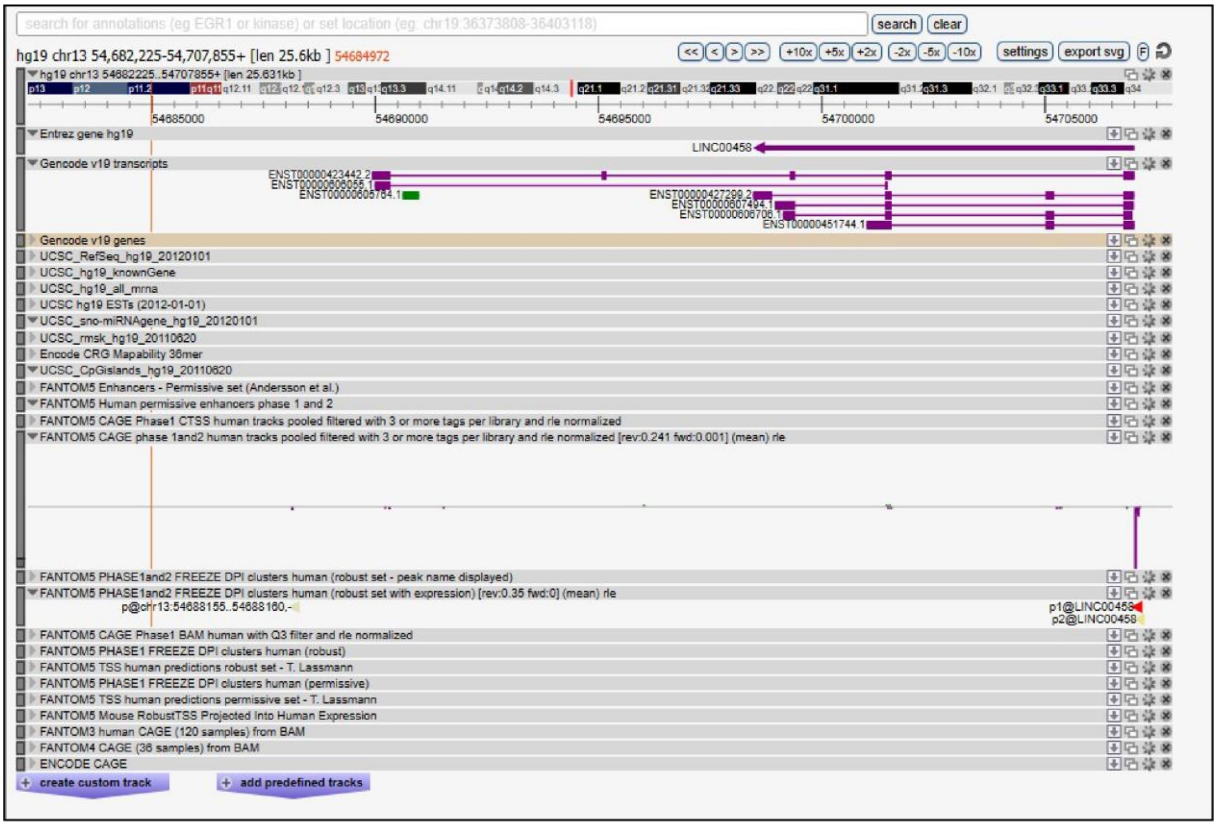

SUPPLEMENTARY FIGURE S7. Transcription start site (TSS) of *LINC00458/HBL1*. Screen shot from ZENBU Genome Browser database (https://fantom.gsc.riken.jp) presenting *LINC00458* TSS information from FANTOM5 CAGE data.

SUPPLEMENTARY FIGURE S8

A

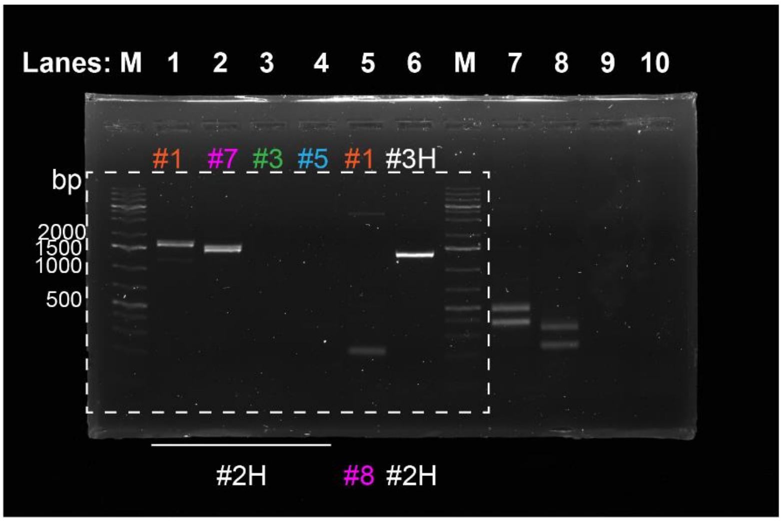

B

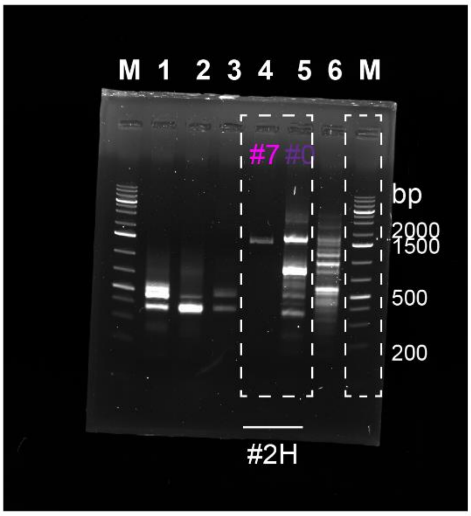

SUPPLEMENTARY FIGURE S8. (**A**) Raw image of the agarose gel presented in Figure 4B. The area delineated by the dashed line is incorporated in Figure 4B. Lanes numbered 7 – 10 are not related to the current manuscript. (**B**) Raw image of the agarose gel presented in Supplementary Fig. S6B. The areas delineated by the dashed line are incorporated in Supplementary Fig. S6B. Lanes numbered 1 – 3 and 6 are not related to the current manuscript (bp = base pairs; M = marker).
